# Development, optimization and microbial stability of innovative gluten-free cereal bars

**DOI:** 10.3389/fnut.2026.1820013

**Published:** 2026-06-15

**Authors:** Sabrina Ferradji, Hayat Bourekoua, Fairouz Djeghim, Radia Ayad, Rayene Belmouloud, Agnieszka Wójtowicz, Renata Rózyło, Adriana Paucean, Luca Masiello, Paola Russo, Giuseppina Adiletta

**Affiliations:** 1 Equipe FNPAA, Laboratoire de Nutrition et Technologie Alimentaire (L.N.T.A.), Institut de la Nutrition, de l'Alimentation et des Technologies Agro-Alimentaires (I.N.A.T.A-A.), Constantine 1, Frères Mentouri University„ Constantine, Algeria; 2 Laboratory of Phytochemistry and Pharmacology, Faculty of Exact Sciences and Informatics, University of Jijel, Jijel, Algeria; 3 Department of Thermal Technology and Food Process Engineering, University of Life Sciences in Lublin, Lublin, Poland; 4 Department of Food Engineering and Machines, University of Life Sciences in Lublin, Lublin, Poland; 5 Faculty of Food Science and Technology, University of Agricultural Sciences and Veterinary Medicine, Cluj-Napoca, Romania; 6 Department of Chemical Engineering Materials Environment Sapienza University of Rome, Rome, Italy

**Keywords:** cereal bars, cold plasma treatment, optimization, pomegranate molasses, sunflower butter, wild seeds

## Abstract

**Background:**

Gluten-free products are frequently produced from unflavored formulas, resulting in poor quality. Individuals with celiac disease may require dietary supplementation with nutritional and bioactive ingredients to rectify deficits and enhance the quality of final products. This study aimed to develop and optimize gluten-free cereal bars using wild seeds (pumpkin, Aleppo pine, and milk thistle seeds), puffed rice, and binders (pomegranate molasses and sunflower butter), and apply cold plasma treatment to assess the effect on the microbial stability.

**Methods:**

A three-simplex centroid mixture design was employed, integrating sensory evaluation and texture parameters. The optimal cereal bars were selected and further evaluated by their physico-chemical and antioxidant properties. Cold plasma treatment was applied to optimized bars, and microbial tests were conducted.

**Results:**

The main results indicated that the combination of 44.8485 g of seeds, 20.1515 g of puffed rice, and 35 g of binder was optimal (optimal 1), followed by the second optimal combination of 20.2049 g of puffed rice, 42.1433 g of seeds, and 37.6518 g of binder (optimal 2). Both cereal bars showed high protein (18.55 and 17.55 g/100 g), fat (25.7 and 30.55 g/100 g), fiber (29.2 and 17.9 g/100 g), and caloric values (422.3 and 416.7 kcal/100 g), respectively. Furthermore, both cereal bars demonstrated high antioxidant activities, with total polyphenol values exceeding 11 mg/g d.w. Texture characterization revealed that both cereal bars exhibited high values of hardness and chewiness with a high overall acceptance rating (7.24 and 7.21, respectively). Cold plasma at 40 W for 20 min reduced microbial loads (over 70%), with Optimal 2 showing the lowest counts (1.84 log CFU/g). During 14 days of storage at room temperature, microbial levels stayed stable and safe, while yeast and mold remained undetectable.

**Conclusion:**

The developed products in this study can be recommended as nutritious, gluten-free, high-energy, and high-fiber bars, and cold plasma proved effective for safety and shelf-life extension.

## 1 Introduction

Celiac disease is an autoimmune disorder triggered by gluten. The primary treatment for celiac disease is a gluten-free diet; however, this diet often fails to adequately address nutritional deficiencies. Therefore, the inclusion of naturally nutrient-dense foods is recommended to improve overall nutritional quality (1–3). In recent years, food consumption patterns have shifted towards healthy, natural, and convenient options. Cereals, especially in forms like ready-to-eat items and cereal bars, have become essential to modern lifestyles (4, 5). Cereal bars, in particular, are gaining popularity as an alternative food source due to their high protein content, bioactive components, and significant energy contribution on a daily basis (6, 7). Market demand for gluten-free products is growing, driving innovation in ingredients and flavors. The global market, especially strong in North America and Europe, faces challenges in developing countries, including product shortages and limited availability. Factors like higher prices, fewer options, and negative consumer reception complicate adherence to gluten-free diets in these regions (94).

According to a recent market analysis, one out of every five consumers consumed cereal bars in 2020. It is predicted that between 2023 and 2028, this market will have a compound annual growth rate (CAGR) of 4.45% globally (8). Commercially available cereal bars exhibit considerable variability in nutritional quality depending on their formulation. Although they are frequently promoted as nutritious snacks, many products have relatively low amounts of protein and dietary fiber and significant amounts of added sugars and fats; consequently, they have high glycemic indices. This problem is particularly relevant in gluten-free products, which are frequently made with refined components and glucose syrup, and may have low quantities of protein, dietary fiber, and key micronutrients as well as reduced levels of iron, calcium, zinc, and B-vitamins compared to conventional cereal-based products (9–11). On the other hand, formulations that include whole grains, dried fruits, seeds, or plant-based proteins typically have better nutritional profiles, especially when it comes to fiber, micronutrients, and bioactive substances. This variation emphasizes the necessity of creating cereal bars that are nutritionally balanced and have better functional and health-promoting qualities (8, 12).

Food experts are actively interested in developing cereal bars enhanced with health-boosting elements. The incorporation of seeds into the human diet contributes to improved nutritional status and reduced risk of chronic diseases (13). In this work, pumpkin seeds have received a lot of attention due to their nutritional value, which includes high levels of protein, fat, carbohydrates, unsaturated fatty acid, fiber, essential minerals such as magnesium, zinc, and iron and phenolic compounds known for their antioxidant properties, which may reduce the risk of degenerative diseases (14, 15). Studies have increasingly explored the incorporation of pumpkin seeds into snack bars (16, 17).

To further mitigate the deficiency of protein and essential fatty acids, milk thistle, Aleppo pine, and sunflower seeds were included. Milk thistle seeds, in particular, characterized by a high oil content and are typically known for their flavonolignans (silymarin complex), which exhibit a strong antioxidant and protective biological activities (18, 19). Aleppo pine seeds may provide an additional nutritional contribution due to their lipid fraction rich in unsaturated fatty acids and their content of bioactive phytochemicals (20). Also, sunflower seeds have a balanced amino acid profile with high glutamic acid, aspartic acid and arginine, and low anti-nutritional factor, rich in unsaturated fatty acids, mainly linoleic and oleic acids and present a high amount of vitamin E, B-vitamins (B_1_, B_5_, folate, niacin), and minerals including selenium, magnesium, phosphorus, copper, manganese, iron, and zinc (21, 22). Furthermore, pomegranate molasses, which used as a natural binder, contains a potential source of polyphenols and organic acids, which may enhance the antioxidant potential of the final product (23, 24).

Despite the absence of prior research on these ingredients in snack bars, their nutritional benefits make them interesting components in nutritious cereal bar formulations. Furthermore, although individual seeds have been examined in snack formulations, the synergistic integration of these particular wild seeds with pomegranate molasses signifies an innovative method for gluten-free enhancement in terms of sensory and functional quality.

Preserving food from microbial contamination is crucial for safety and extending shelf life. Traditionally, food preservation focuses on physical and biological methods such as fermentation, sun drying, salting, and roasting (25). However, advances in food processing technology have led to the development of innovative non-thermal preservation methods, to keep the bioactive chemicals in these seeds that are sensitive to heat, among which cold plasma has gained increasing attention. Cold plasma (CP) is considered an environmentally friendly and energy-efficient technology, compatible with a wide range of food products (26, 27). Recent studies have demonstrated the effectiveness of cold plasma in reducing microbial load and extending the shelf life of foods while maintaining product quality (25, 28, 29). Even though it has promise, there isn't much information in the literature about using cold plasma on cereal bar matrices with a lot of different ingredients.

The efficiency of the cold-plasma process strongly depends on treatment factors such as gas composition, exposure duration, voltage, and product attributes. Furthermore, adverse changes, including lipid oxidation, bioactive component degradation, structural changes to macromolecules, or changes in sensory qualities, might be brought on by excessive or poorly managed treatment circumstances (30, 31).

Although the incorporation of seeds into cereal bars has been widely investigated, most studies focus on commonly used seeds such as chia, flax, and sunflower, and do not explore combinations with underutilized functional seeds (7, 32–34). Accordingly, the aim of this research was to formulate and optimize innovative, nutritious, and distinctive multi-seed gluten-free bars using combinations of puffed cereals, wild seeds and a natural binder. Furthermore, the resulting bars were subjected to cold plasma treatment to evaluate the impact of this non-thermal technology on the microbial stability of the final products. The focus of this study was on creating cereal bars with optimal technological and sensory characteristics, high antioxidant capacity, and extended shelf life to meet the nutritional needs of consumers with celiac disease. A three-simplex centroid mixture design was employed to achieve the desired formulations.

## 2 Materials and methods

### 2.1 Chemical

The chemicals used in this study were supplied by Sigma-Aldrich, Steinheim, Germany, and include: 1,1-diphenyl-2-picrylhydrazyl (DPPH), 2,2′-azino-bis (3-ethylbenzothiazoline-6-sulfonic acid) (ABTS), ammonium molybdate, Folin–Ciocalteu reagent, ferric chloride (FeCl_3_) potassium ferricyanide K_3_[Fe(CN)_6_], trichloroacetic acid (TCA). As standards: ascorbic acid, gallic acid, quercetin. The solvents, ethanol and methanol, were supplied by Sigma-Aldrich (St. Louis, MO, USA). All reagents and solvents used were of analytical grade.

### 2.2 Raw materials

Pumpkin seeds (*Cucurbita maxima*), Aleppo pine seeds (*Pinus halepensis*), milk thistle seeds (*Silybum marianum*), and sunflower seeds (*Helianthus annuus*) were purchased from a local market in Algeria. Whole seeds were used in this study. Pumpkin seeds contained 6.06% moisture, 36.92% protein, 41.2% fat, and 5.06% ash. Aleppo pine seeds had 6.65% moisture, 23.55% protein, 29.8% fat, and 6.76% ash. Milk thistle seeds had 7.25% moisture, 16.66% protein, 19.3% fat, and 3.75% ash. Sunflower seeds contained 6.93% moisture, 30.53% protein, 46.3% fat and 3.53% ash (dry matter).

Pomegranate seeds (*Punica granatum*), containing 23.10% moisture, 1.7% protein, 3.7% fat, and 0.44% ash, were purchased from a local market in Algeria. Rice grains (11.8% moisture, 6% protein, 0.21% fat 1.66% ash) were provided by Hawas Food Company (Alger, Algeria).

### 2.3 Preparation of raw materials

#### 2.3.1 Pomegranate molasses

Pomegranate molasses (moisture content of 23%, pH-value of 4.37, refractive index of 1.48, soluble solid content of 79.13° brix, and kinematic viscosity of 3.976 × 10^4^ m^2^/s) was prepared according to Abdelkhalek (35). Approximately 2 kg of pomegranate seeds were washed, peeled, juiced, and filtered. The juice was then thermally treated to around 200 °C and stirred continuously for about 15 min until thickened into molasses.

#### 2.3.2 Puffed rice

Rice grains were puffed (about 10 s) in the presence of hot oil at 200–220 °C, following the method described by Bourekoua et al. (7).

#### 2.3.3 Sunflower butter

Sunflower seeds were roasted at 160 °C for 20 min, shelled, and then mashed into natural butter using a high-speed grinder (BRANDMANN 700W, China). The butter, containing no additives, was stored at 4 °C.

### 2.4 Gluten-free cereal bar formulation

The formulation investigated in this study was based on puffed rice and a mixture of wild seeds (pumpkin, Aleppo pine, and milk thistle seeds) in equal quantities. As binders, pomegranate molasses and sunflower butter were combined in a ratio of 5/3 for each formulation.

#### 2.4.1 Experimental design

A three-simplex centroid mixture design augmented with 10 formulations was employed to optimize the proportions of the three ingredients employed in bar formulations.

The findings allowed for the determination of the regression coefficients, and each response was modeled as a function of coded components as follows:


$$\begin{array}{l} Y=\overset{3}{\underset{i=1}{\sum }}\;\alpha_{i}X_{i\;}+\sum \overset{3}{\underset{i<j}{\sum }}\alpha_{ij}X_{i}X_{j}+\sum \sum \overset{3}{\underset{i<j<k}{\sum }}\alpha_{ijk}X_{i}X_{j}X_{k} \end{array}$$

Where Y is the expected response value; X_1_, X_2_, and X_3_ are independent components (puffed rice as cereals, mixed seeds (pumpkin, Aleppo pine, and milk thistle seeds), and binders (pomegranate molasses and sunflower butter); *i* is the linear effect, and *ij* and *ijk* are the interaction effects. The preliminary testing identified the maximum and lowest amounts of the variables (puffed rice: 15–25 g, seeds: 40–50 g, and binder: 35–45 g). Formulations of cereal bars according to the mixture design matrix are shown in Table 1.

**Table 1:** Mixture design of the cereal bars formulations.

Formulation	Puffed rice (g)	Seeds (g)	Binder (g)	Total (g)
1	18.3333	43.3333	38.3334	100
2	15.0000	40.0000	45.0000	100
3	15.0000	50.0000	35.0000	100
4	16.6666	46.6667	36.6667	100
5	25.0000	40.0000	35.0000	100
6	20.0000	45.0000	35.0000	100
7	16.6666	41.6667	41.6667	100
8	20.0000	40.0000	40.0000	100
9	15.0000	45.0000	40.0000	100
10	21.6666	41.6667	36.6667	100

The responses Y were based on the sensory characteristics and textural parameters of the gluten-free cereal bars, which are translated by taste (Y_1_), appearance (Y_2_), crispness (Y_3_), overall acceptability (Y_4_), chewiness (Y_5_) and hardness (Y_6_).

The optimization was conducted using the desirability function, which represents a statistical tool intended to concurrently optimize numerous response variables by integrating them into a singular composite desirability score. Each response variable is scaled from 0 (di = 0, representing the least favorable outcome) to 1 (di = 1, representing the most favorable outcome) according to established standards.

#### 2.4.2 Preparation of gluten-free cereal bars

Gluten-free cereal bars were made using the process described by Bourekoua et al. (7) with slight modifications. The preparation procedure involved combining three components (puffed rice, seeds, and binder) based on the mixture design (Table 1). All components were hand-blended in a container.

Hand-blended technique is chosen based on the physical properties of the bar ingredients, specifically puffed rice and wild seeds, which can be physically damaged by mechanical mixing due to shear stress, thereby affecting the texture of the bars. Manual mixing helps preserve the integrity of the ingredients. To ensure homogeneity of the mixture, the dry ingredients were weighed precisely according to different formulations, and pre-mixed carefully for 1 min using light, circular motions to ensure better blending of the ingredients before adding the binding agent. The binder, consisting of sunflower butter and pomegranate molasses, was heated and mixed beforehand at a temperature of 35 °C to reduce viscosity and to ensure a uniform consistency, then gradually added to the mixture of dry ingredients while mixing continuously and thoroughly until a well-bound bar mass was obtained. A standardized “folding and turning” technique was used at a constant rate of 25 to 25 movements per minute. This involves scraping the spatula from the bottom of the container, lifting the dough mass, and folding it back toward the center to ensure three-dimensional distribution without crushing the puffed rice or seeds; this process lasted 5 min for all formulations. This time was determined based on achieving a glossy, uniform coating appearance for the formulations. The resulting bar mass was divided into 30 g pieces and placed in a mold (9 cm × 3 cm × 2.5 cm). To ensure stability, the cereal bars were stored at 4 °C for 15 min before analysis.

### 2.5 Evaluation of cereal bars properties

#### 2.5.1 Textural analysis

The texture properties of cereal bars were evaluated using a Zwick/Roell type BDOFBO.5TH testing machine (Zwick GmbH & Co., Ulm, Germany) equipped with an OTMS Ottawa cell. Bars (2.5 cm × 2.5 cm × 2.5 cm) underwent a double compression test up to 75% of their original height at a cross-head speed of 3 mm/s, with a 15-s interval between compressions. TestXpert^®^ 13.3 software (Zwick GmbH & Co., Ulm, Germany) recorded and analyzed hardness and chewiness in three replications. Hardness was defined as the force required, in a first compression cycle, to induce deformation until the sample was snapped (36), chewiness = hardness × springiness × cohesiveness and represents the force required to chew (disintegrate) products (37).

#### 2.5.2 Proximate composition and calorific value

To determine the proximate composition of cereal bars, the samples were dried at 40 °C and subsequently ground into a fine powder. The AOAC methods were used: the 42.05 method for ash and the 960.52 method for protein content, and the 996.01 method for fat content (38). The insoluble, soluble, and total dietary fiber content were determined following the AACC 991.43 method (39). Moisture content was determined using the method of ICC 110/1 (40). By deducting the amounts of protein, fat, moisture, ash, and dietary fiber from 100% of the dry matter, the amount of carbohydrates was calculated. The calorific value (kcal per 100 g of bars) was estimated using the following factors: 9 for fats, 4 for carbohydrates, and 4 for proteins (41). Analysis were conducted in three replicates.

#### 2.5.3 Antioxidant properties

##### 2.5.3.1 Ultrasound-assisted extraction process

The extraction procedure was carried out following Ayad et al. (42). One gram of dried cereal bar powder was mixed with 10 ml of 80% ethanol and subjected to ultrasound-assisted extraction using ultrasonic cleaning bath equipment (ultrasons-H, 50/60 Hz, 720 W, Ctra. Nll Km: 585.1 Abrera, Barcelona, Spain). The sonication was performed for 60 min at room temperature. After extraction, the samples were chilled and then filtered through Whatman No. 1 paper for further analysis.

##### 2.5.3.2 Total phenolic content

The Folin–Ciocalteu method was used as described by Singleton and Rossi (43). It was utilized to assess the total phenolic content, which was then quantified as gallic acid equivalents (GAE, mg/g d.w.). A 0.30 ml extract sample was combined with 1.20 ml of the diluted Folin-Ciocalteu reagent (1:10). After 5 min, 1.50 ml of a 7.50% Na_2_CO_3_ solution was added to the combination, which was then allowed to rest at room temperature in the dark for 2 h. A UV/visible spectrophotometer (Thermo Electron Corporation Evolution 100) was used to detect the absorbance at 765 nm. The TPC was calculated using the calibration curve, which was created by using a gallic acid solution (0–200 μg/ml). The findings are shown as mg GAE/g d.w. Three replicates of the experiment were carried out.

##### 2.5.3.3 Total flavonoid content

The total flavonoid content of the powdered cereal bars was determined according to Djeridane et al. (44). One 1 ml of the extract was combined with an equal volume of a 2% AlCl_3_ solution. After 10 min of incubation at room temperature, the absorbance was measured by the spectrophotometer (Thermo Electron Corporation Evolution 100) at 430 nm. The total flavonoid content was expressed in milligrams of quercetin equivalent per gram of dry weight (mg QE/g d.w.). Analysis were performed in three replicates.

##### 2.5.3.4 Antioxidant activity

The antioxidant activity (AA) of cereal bar extracts was assessed using four techniques, three replicates each, including total antioxidant capacity (TAC) (45), DPPH radical scavenging activity (46), ABTS radical scavenging assay (47), and ferric reducing antioxidant power (FRAP) (48). Total antioxidant activity (TAC) was measured in milligrams of ascorbic acid equivalent per gram dry weight (mg AAE/g d.w.), DPPH and ABTS scavenging activities were expressed as EC_50_ value in mg d.w./ml (the concentration providing 50% inhibition), and ferric reducing antioxidant power (FRAP) was recorded as an A_0.5_ value corresponding to the absorbance of 0.5 that was calculated from the graph plotting absorbance against sample concentration.

#### 2.5.4 Sensory evaluation

Sensory characteristics of cereal bars were assessed. Fifty five tasters (participated in a consumer-based acceptability test using a nine-point hedonic scale: 1 (dislike extremely), 5 (neutral), and 9 (like extremely). Panelists included non-smokers, and experienced food tasters who participated voluntarily. Taste, crispness, appearance, and overall acceptability were recorded (49). The selected panelists gave their consent to participate. Prior to the test, participants received an explanation of the study's objective, and they provided their consent in compliance with the INATAA Institute's ethical procedures, which are based at Constantine 1 Frères Mentouri University.

#### 2.5.5 Water activity

Water activity was measured using a digital humimeter (Humimeter RH2, Schaller, Austria). The samples and the instrument were adjusted to the surrounding temperature (25 °C ± 1) for at least 30 min. Sample bars (2.5 g) was placed in a thermostatic cell at 25 °C, and the measure was carried out after 10 min in three replicates.

### 2.6 Cold Plasma sanitization treatment and microbial evaluation

Cold plasma treatment was performed using a Dielectric Barrier Discharge (DBD) system (Redline Technologies, Elektronik GmbH, Baesweiler, Germany) equipped with a High Voltage Plasma Generator G2000 (voltage range 0–30 kV; frequency range 3.3–500 kHz). The apparatus is installed in the laboratories of Prof. Russo and Dr. Adiletta at the Department of Chemical Engineering, Materials and Environment, Sapienza University of Rome. The system consisted of a Plexiglas chamber (30 × 30 × 30 cm3) with a wall thickness of 2.5 cm, containing two parallel steel electrodes (15 × 15 cm^2^, thickness 2 mm) acting as the dielectric barrier. The upper electrode position was manually adjustable in order to control the inter-electrode distance.

For these preliminary trials, cereal bars were placed in a glass Petri dish (2 mm thickness) positioned between the two electrodes. Samples were treated at a power of 40 W, a voltage of 20 kV, a frequency of 71 kHz, and a current of 0.20 A for 20 min, while the electrode spacing was adjusted to obtain a uniform plasma distribution over the sample surface. These operating conditions were selected according to the optimized parameters reported by Suhem et al. (50), who investigated cold atmospheric plasma for the inhibition of Aspergillus flavus on brown rice cereal bars. Their results showed that treatment at 40 W for 20 min effectively delayed fungal growth during storage. Considering the different food matrix of the present study, the same exposure time was adopted as a suitable compromise to ensure microbial control while minimizing potential quality alterations in the treated samples. Power, frequency, and voltage were continuously monitored and adjusted to ensure stable plasma generation throughout each treatment cycle.

Microbiological analyses were performed to assess the total microbial load in terms of aerobic mesophilic bacteria, yeasts, and molds in the treated and untreated bars. Approximately 5 g of each sample were aseptically collected and transferred into sterile Stomacher bags containing 45 ml of sterile 0.9% (w/v) NaCl solution (51). Samples were homogenized for 2 min using a Stomacher 400 Circulator (Seward, England) to obtain a uniform microbial suspension. Serial decimal dilutions were subsequently prepared in sterile saline solution. The total viable count was determined on 3M^®^ Petrifilm Aerobic Count Plates, whereas yeasts and molds were enumerated on 3M^®^ Petrifilm Yeast and Mold Plates. All plates were incubated at 30 °C for 24–48 h. Microbial results were expressed as the logarithm of colony-forming units per gram of sample (log CFU/g). Each biological replicate was analyzed in triplicate to ensure methodological robustness and data reliability.

#### 2.6.1 Statistical analysis

The experimental design and analysis were conducted using Minitab statistical software, version 19 (Minitab Inc., State College, PA, USA). Means were compared and analyzed using one-way analysis of variance (ANOVA), followed by Tukey's significant differences *post hoc* test, all performed with JMP 17 (SAS Institute Inc, Cary, US). The two optimal formulations were compared using a *t*-test. A statistical difference was considered significant when *p* < 0.05.

## 3 Results

### 3.1 Mixture design analysis and model fitting

All of the models examined in this study had acceptable coefficients of determination and predicted R-squared values (Table 2). In the context of cereal bar evaluations, R^2^-seq variations spanned from 70% for crispness to 95% for hardness.

**Table 2:** Regression coefficient of the gluten-free cereal bars responses with predicted R-square values.

Response	Taste		Appearance		Crispness		Overall acceptability		Chewiness		Hardness	
	Coeff	*P* value	Coeff	*P* value	Coeff	*P* value	coeff	*P* value	Coeff	*P* value	Coeff	*P* value
Puffed rice	−304.9	< 0.05^*^	−116.5	< 0.05^*^	−23.1	< 0.05^*^	−295.1	< 0.05^*^	−678	< 0.05^*^	885	< 0.05^*^
Seeds	−42.6	< 0.05^*^	−0,9	< 0.05^*^	97.4	< 0.05^*^	−11.2	< 0.05^*^	395	< 0.05^*^	−962	< 0.05^*^
Binder	−16	< 0.05^*^	−14/8	< 0.05^*^	140.3	< 0.05^*^	−12,4	< 0.05^*^	217	< 0.05^*^	−2689	< 0.05^*^
Puffed rice × seeds	593	0,016	179	0.171	11	0.950	463	0.041	−141	0.889	−3969	0.272
Puffed rice × binder	379	0.063	229	0.100	−144	0.445	469	0.039	1565	0.173	5263	0.016
Seeds × binder	81	0.612	23	0.838	−454	0.056	9	0.958	−1321	0.235	8974	0.045
R-square	86.47%	79.55%	70.90%	82.02%	84.00%	95.75%

^*^Significant value in either magnitude or probability (p < 0.05).

Prior to process optimization, the regression coefficients of component effects were evaluated. Table 2 presents the regression coefficient, which translates the dependence of the response to the components of the mixture. According to the results, the linear effects of the three components and two-way interaction (puffed rice × seeds) in taste and overall acceptability were significant (*p* < 0.05), but the two-way interactions puffed rice × binder and seeds × binder showed non-significant effects (*p* > 0.05) for taste, appearance, crispness, hardness and chewiness response. The two-way interactions puffed rice × binder and seeds × binder present a significant effect for hardness and overall acceptability.

### 3.2 Effect of the mixture's components on the sensorial and textural parameters

Sensorial characteristic ratings and textural properties of cereal bars according to mixture design formulations are shown in Table 3.

**Table 3:** Experimental results for taste, appearance, crispness, overall acceptability, chewiness, and hardness of the different gluten-free cereal bar formulations.

Formulation	Taste	Appearance	Crispness	Overall acceptability	Chewiness	Hardness
1	7.00 ± 1.414^a^	6.53 ± 1.595^a^	7.00 ± 1.38^a^	7.07 ± 1.382^a^	6.36 ± 0.979^e^	262.25 ± 4.242^ab^
2	6.00 ± 0 .951^a^	5.84 ± 1.618^a^	7.84 ± 1.519^a^	6.7 ± 0.948^a^	12.61 ± 0.02^g^	274.00 ± 2.828^ab^
3	5.92 ± 1.61^a^	6.46 ± 1.831^a^	7.92 ± 1.44^a^	6.53 ± 1.290^a^	4.04 ± 1.90^f^	258.00 ± 0.707^ab^
4	7.00 ± 1.779^a^	6.38 ± 1.757^a^	8.00 ± 1.224^a^	7.61 ± 1.325^a^	9.74 ± 1.07^d^	267.00 ± 4.242^ab^
5	5.00 ± 1.37^a^	6.38 ± 1.361^a^	7.30 ± 1.436^a^	6.84 ± 1.463^a^	2.915 ± 1.43^g^	214.2 ± 16.97^b^
6	6.69 ± 1.634^a^	6.92 ± 1.552^a^	7.53 ± 1.560^a^	7.28 ± 0.755^a^	11.28 ± 9.470^c^	275.6 ± 3.535^ab^
7	6.076 ± 1.712^a^	6.461 ± 1.213^a^	7.00 ± 1.04^a^	7.181 ± 1.470^a^	19.79 ± 0.33^a^	231.5 ± 7.071^ab^
8	6.307 ± 1.577^a^	6.692 ± 1.337^a^	7.230 ± 1.42^a^	7.454 ± 1.293^a^	12.162 ± 0.11^b^	291.6 ± 15.55^a^
9	6.00 ± 1.728^a^	6.307 ± 1.489^a^	6.615 ± 1.80^a^	6.615 ± 1.605^a^	9.5775 ± 0.94^d^	287.875 ± 9.89^ab^
10	6.615 ± 1.505^a^	7.153 ± 1.302^a^	6.923 ± 1.55^a^	7.538 ± 1.450^a^	10.14 ± 0.26^d^	239.875 ± 6.363^ab^

^*^Different superscript letters a–e at each column indicates significant differences (p < 0.05).

Table 3 displays the experimental findings from the mixture design study. According to the results, it can be stated that there was no significant difference in the hardness values across all experiments. F8 and F9 exhibit higher values with 291.6 N and 287.875 N, respectively. The lowest value was observed for F5 (214.2N).

For chewiness, there were significant differences (*p* < 0.05) among experiments. The highest value of chewiness was observed for F7 with a value of 19.79 N, which contains 41.66 g of seeds, and binder, and the lowest value of chewiness was observed for F5 with 2.92 N. Concerning the other sensorial attributes–taste, appearance, and overall acceptability–there was no significant statistical difference (*p* < 0.05) between formulations according to consumer appreciation.

All cereal bars exhibited good sensory acceptance for all the parameters analyzed. The average values of most attributes varied between 6 and 8. The differences in tasters' ratings are caused by particular preferences for the product under discussion.

**Figure 1:**
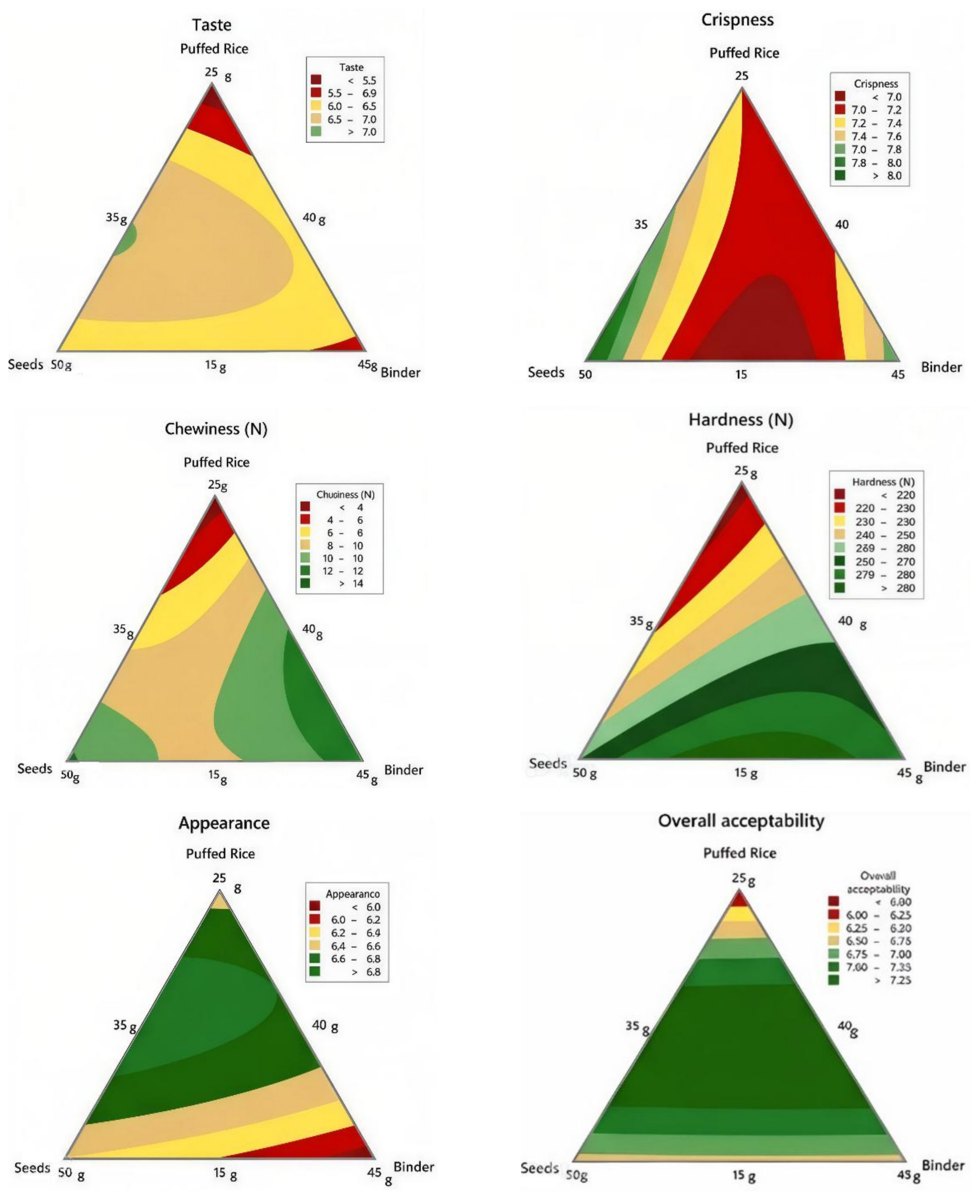
Contour plots representing the effect of puffed rice, seeds, and binder on the predicted sensory attributes of gluten-free cereal bars: **(A)** taste, **(B)** crispness, **(C)** chewiness, **(D)** hardness, **(E)** appearance, and **(F)** overall acceptability.

The contour plots (Figure 1) indicate that hardness (Figure 1D) increases as the proportion of puffed rice decreases and the proportions of seeds (50 g w/w) and binder (45 g w/w) increase. The lowest hardness values (220–230 N) were found with higher puffed rice content. The same observation was shown for chewiness, with the lowest values located in the region with higher puffed rice. Otherwise, the highest values of chewiness (Figure 1C) were located in the region with maximum binder (around 45 g). Seeds exhibit both impacts on chewiness, which translate to maximum chewiness in the region of lower and higher seeds.

Concerning taste (Figure 1A), the lowest score was observed around 25 g of puffed rice and 45 g of binder, while the optimal zone was located around 35 g of binder, 15 g of puffed rice and 50 g of seeds. The lowest scores of crispness (Figure 1B) were located in the region with lower puffed rice, around 15 g w/w. An increasing trend was identified until the highest level of puffed rice with scores of taste from 7 to 7.2. The optimal zone was located in the region with the minimum level of binder and the maximum level of seeds (about 50 g w/w).

Regarding appearance and overall acceptability (Figures 1E, 1F respectively), it was assumed that a large optimal zone was observed around 40 g (w/w) of seeds and a minimal amount of binder. The lowest score for appearance was noted for the maximum amount of binder and the minimum level of puffed rice; for the overall acceptability, the lowest score was noted for the maximum amount of all ingredients (puffed rice, binder, and seeds).

### 3.3 Optimization results

A multicriteria optimization study using the desirability function aimed to maximize sensory attributes (taste, crispness, appearance, overall acceptability) while minimizing hardness and chewiness. The optimal formulation, containing 44.85 g of seeds, 20.15 g of puffed rice, and 35 g of binders, achieved the highest desirability value (D = 0.81). Another formulation, with 20.20 g of puffed rice, 42.14 g of seeds, and 37.65 g of binders, had a lower but acceptable desirability value (D = 0.68) but minimized crispiness.

#### 3.3.1 Characteristics of optimal gluten-free cereal bars

##### 3.3.1.1 Textural properties

Table 4 shows the texture criteria for the two optimal cereal bars. The results presented in Table 4 indicate no significant difference in hardness (*p* > 0.05) between the two optimal cereal bars, with both exhibiting values exceeding 225 N. Both bars also demonstrated high chewiness.

**Table 4:** Textural parameters of optimal cereals bars.

Sample	Textural parameters	
	Hardness (N)	Chewiness (N)
**Optimal 1**	227.4 ± 9.192^a^	7.24 ± 1.105^a^
**Optimal 2**	243.8 ± 5.655^a^	8.06 ± 0.608^a^

##### 3.3.1.2 Biochemical composition and calorific value

The chemical composition and calorific values of the optimal cereal bars are presented in Table 5.

**Table 5:** Chemical composition and caloric value of optimized cereal bars.

Sample	Moisture (%)	Protein (%)	Fat (%)	Ash (%)	Fiber (%)			Carbohydrate (%)	Caloric value (kcal/100 g)
					Soluble	Insoluble	Total fiber		
Optimal 1	8.51 ± 0.110^a^	18.55 ± 0.086^a^	25.70 ± 0.003^b^	3.24 ± 0.111^b^	2.91 ± 0.01^b^	20.41 ± 0.015^b^	23.31 ± 0.37^b^	29.20 ± 0,002^a^	422.30 ± 0.010^a^
Optimal 2	8.77 ± 0.360^a^	17.55 ± 0.154^a^	30.55 ± 0.001^a^	4.36 ± 0.010^a^	7.96 ± 0.020^a^	21.67 ± 0.003^a^	29.64 ± 0.650^a^	17.90 ± 0,010^b^	416.50 ± 0.020^a^

^a − b^-different letters in columns indicate significant differences at 0.05.

The results of the chemical composition and caloric value analysis of the two optimal cereal bars, presented in Table 5, indicate no significant difference in moisture content (*p* > 0.05), with values of 8.51% and 8.77%. Similarly, protein content did not vary significantly, with values of 18.55 g/100 g and 17.55 g/100 g. However, a significant difference (*p* < 0.05) was observed in fat content, where the second bar contained a higher fat level (30.55 g/100 g) compared to the first (25.70 g/100 g). The ash content, representing the mineral composition, was also significantly greater in the second bar (4.36 g) than in the first (3.24 g). Fiber content showed notable variation, with the second bar containing 29.64 g compared to 23.31 g in the first cereal bar. Conversely, the first bar contained a higher carbohydrate content (29.2 g) compared to the second (17.9 g). Caloric analysis revealed the first bar to be more energy-dense at 422.30 kcal/100 g, surpassing the caloric value of the second bar at 416.50 kcal/100 g.

##### 3.3.1.3 Antioxidant properties

Regarding the antioxidant activities (Table 6), overall, optimal 2 exhibited the highest total phenolic content and the best antioxidant activities with TAC, DPPH, and FRAP, followed by the first optimal. The highest value of TPC was 11.688 GAE mg/g d.w., slightly higher than the optimal 1 (11.196 GAE mg/g d.w.). In contrast, the TFC was higher in optimal 1 with 0.647 QE mg/g d.w., compared to optimal 2. TAC of optimal 2 presents high value 2.574 AAE mg/g d.w., compared to the first optimal 2.205 AAE mg/g d.w., a similar trend was observed for the FRAP assay, where optimal 2 present the strongest reducing capacity, 3.377 mg d.w./ml compared with 2.788 mg d.w./ml. For DPPH, optimal 1 present higher activity with an IC_50_ of 1.149 mg d.w./ml which is lower than the second optimal 1.747 mg d.w./ml. Conversely, ABTS results show that optimal 1 had the highest IC_50_ value, at 1.137 mg d.w./ml, indicating its low antioxidant activities. The high antioxidant activity observed in the two optimal cereal bars can be directly linked to the rich composition of bioactive compounds in the ingredients used.

**Table 6:** Antioxidant properties of the two optimal cereal bars.

Sample	TPC (GAE mg/g d.w.)	TFC (QE mg/g d.w.)	TAC (AAE mg/g d.w.)	DPPH: IC_50_ (mg d.w./ml)	ABTS:IC_50_ (mg d.w./ml)	FRAP:A_0, 5_ (mg d.w./ml)
Optimal 1	11.196 ± 0.025^a^	0.647 ± 0,120^a^	2.205 ± 0.020^b^	1.149 ± 0.044^b^	1.137 ± 0.137^a^	2.788 ± 0.066^b^
Optimal 2	11.688 ± 0.060^a^	0.421 ± 0,011^b^	2.574 ± 0.015^a^	1.747 ± 0.066^a^	0.351 ± 0.020^b^	3.377 ± 0.015^a^

TPC, Total Phenolic content; TFC, Total Flavonoid content; TAC, total antioxidant capacity, DPPH, ability to quench DPPH radicals; ABTS, ability to quench ABTS radicals; FRAP, Ferric Reducing Antioxidant Power. Values in the same column not sharing same letters are significantly different (P < 0.05).

##### 3.3.1.4 Sensorial characteristics

Sensorial characteristics of the two optimal cereal bars are shown in Table 7.

**Table 7:** Sensory characteristics of the optimal cereal bars.

Parameter	Sensory attributes				
	Taste	Crispness	Aroma	Appearance	Overall acceptability
Optimal 1	6.84 ± 1.120^a^	7.55 ± 0381^a^	5.875 ± 1.497^a^	6.40 ± 0.994^a^	7.24 ± 0.925^a^
Optimal 2	6.61 ± 1.926^a^	7.08 ± 0.472^a^	6.26 ± 1.436^a^	6.80 ± 1.585^a^	7.21 ± 1.257^a^

The sensory evaluation results presented in Table 7 indicate that there was no significant difference between the two cereal bars in terms of taste, crispness, texture, and overall acceptability. Overall, panelists found both cereal bars formulation as acceptable. Both bars received high scores from the panelists, reflecting high consumer acceptance and appreciation of these two innovative products in terms of taste, crispness, and texture. These results thus indicate that both formulations are viable from a sensory perspective, with overall acceptability scores of 7.21 and 7.24 for formulations 1 and 2, respectively.

##### 3.3.1.5 Water activity

The water activity results of the two optimal cereal bars are presented in Table 8. Both formulations showed low water activity with optimal 1 recording 0.483 ± 0.002 and optimal 2 recording 0.481 ± 0.002. These results exhibited highly reproducible water activity (a_w_) values.

**Table 8:** Water activity of the optimal cereal bars.

Parameter	Optimal 1	Optimal 2
Water activity (a_w_)	0.483 ± 0.002	0.481 ± 0.002

#### 3.3.2 Results of microbiological analysis

**Figure 2:**
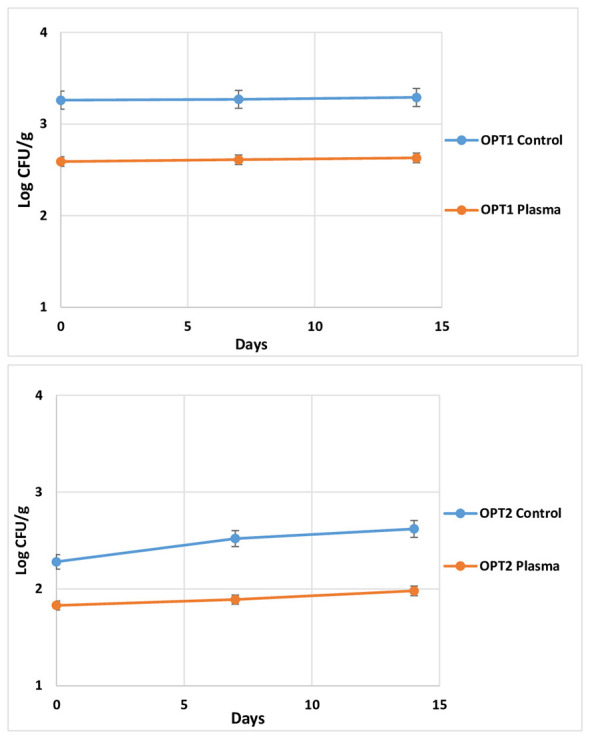
Impact of cold plasma treatment on the microbial load of gluten-free cereal bars. Comparison between untreated (control) and treated samples regarding total aerobic mesophilic bacteria. Opt1: optimal1; Opt 2: optimal 2.

Microbiological analysis including aerobic mesophilic bacteria, yeast and molds of the untreated and treated two optimal bars are shown in Figure 2.

The total microbial counts of untreated and cold plasma-treated cereal bars were assessed using standard plate count methodology at time points 0, 7, and 14 days of storage at room temperature in the original packaging.

For the untreated samples (Figure 2), formulation of optimal 1 showed an initial aerobic mesophilic bacterial count of 3.23 ± 0.10 log CFU/g at day 0, which remained relatively stable at 3.26 ± 0.12 log CFU/g after 7 days and slightly increased to 3.29 ± 0.14 log CFU/g after 14 days. In contrast, formulation of optimal 2 exhibited a lower initial value of 2.48 ± 0.08 log CFU/g at 0, gradually increasing to 2.54 ± 0.10 log CFU/g at day 7, and reaching 2.63 ± 0.11 log CFU/g at day 14. Throughout the storage period, both untreated and treated formulations remained within the acceptable microbiological quality limits for ready-to-eat low-moisture products. Cold plasma treatment at 40 W for 20 min resulted in a marked reduction of the microbial load in both formulations. Treated optimal 1 samples showed an initial value of 2.59 ± 0.09 log CFU/g at day 0, with a slight increase to 2.61 ± 0.10 log CFU/g at day 7 and 2.64 ± 0.12 log CFU/g at day 14. Optimal 2 treated samples demonstrated the most significant reduction, with an initial microbial load of 1.84 ± 0.07 log CFU/g at 0, increasing to 1.89 ± 0.08 log CFU/g at t7, and reaching 1.98 ± 0.10 log CFU/g after 14 days.

## 4 Discussion

### 4.1 Model fitting and effect of components on the sensorial and textural analysis

Concerning the model fitting for the mixture design, the results suggest the appropriateness and representativeness of the chosen model for the system, further validating the established correlation between the selected response and the respective components.

The results indicated an adequate experimental model. As indicated by Bourekoua et al. (52), a positive main effect suggests that a high concentration of a factor is nearly optimal, while a negative main effect indicates that a low concentration is nearly optimal. Based on the tabulated data, it is anticipated that the inclusion of the three components in minimal proportions within the formulation will positively impact the sensory attributes, namely taste, appearance, and overall acceptability, of gluten-free cereal bars. High concentrations of seeds and binder enhance crispness and chewiness, while low quantities increase hardness.

The differences in hardness values among the 10 formulations can be explained by the addition of more seeds, which makes the product harder. This finding is consistent with the research conducted by Manguldar et al. (16), who also observed that higher pumpkin seed concentrations were associated with greater hardness. Furthermore, Muniz et al. (53) suggested that high hardness values might be linked to large quantities of seeds rich in protein and fiber. This conclusion is supported by the research of Rawat and Darappa (54), who found that adding a combination of fiber-rich components to protein bar dough increased the dough's firmness.

According to Bower and Whitten (55), crispness is the most essential factor influencing customer acceptance. The testers appreciate gluten-free bars made from F4 components, followed by F3 and F2 components, which contained more seeds than binder.

The differences in tasters' ratings are likely caused by particular individual preferences for the specific product type. The observed increase in hardness with higher seed concentrations. This aligns with Manguldar et al. (16), who noted that higher pumpkin seed concentration increases hardness. Muniz et al. (53) suggested that high hardness is linked to seeds rich in protein and fiber, supported by Rawat and Darappa (54), who found that fiber-rich components increase dough firmness.

### 4.2 Characteristics of optimal cereal bars

Concerning the optimization process of cereal bar formulation, despite minimizing the crispness, the formulation is still optimal. The comparison of these two optimal bars was conducted to validate the model and offer consumers well-formulated options.

The similarity in texture profile between the two optimal formulations can be attributed to the incorporation of several seeds in the formulation, which are naturally rich in fiber. Carvalho and Conti-Silva (56) confirmed that fiber-rich foods tend to be chewier and harder, aligning with earlier findings by Freitas and Moretti (57) and Samakradhamrongthai et al. (58). From practical perspective, this increased chewiness may represent an advantage for cereal bars intended as satiety-promoting snacks since fiber-rich foods generally provide prolonged mastication and slower digestion (59). Overall, these studies highlight the role of fiber and sugar levels in enhancing the breaking strength and chewiness of food products.

In relation to biochemical composition, the low moisture content observed in the two optimal cereal bar formulations is consistent with the findings of Kaur et al. (60) for cereal bars containing quinoa, flaxseed, and fruit, which recorded a moisture content of 8.53%. This similarity may be attributed to the use of the low-moisture ingredients such as puffed rice and seeds, as well as binding agent rich in sugars and lipids which tend to reduce free water availability in the final formulation. Also its underscores the significance of maintaining low moisture levels to improve sensory qualities like crispness while also emphasizing a strong formulation process that can reduce microbial growth, prevent texture degradation, and avoid sensory deterioration (61). The reduced moisture content in the cereal bars examined in this study suggests a likelihood of longer shelf life and enhanced appeal among consumers.

Regarding the protein, the values were higher than those reported by Manguldar et al. (16) and consistent with that of Sharma et al. (62), supporting the hypothesis that seed percentage significantly increases protein content. The rise in protein levels is linked to the high protein level of seeds particularly pumpkin, milk thistle and sunflower seeds used in the formulation.

The difference in fat content of the two cereal bars is primarily due to high-fat ingredients such as sunflower butter and pumpkin seeds. Pumpkin seeds and sunflower seeds contain 41.20 and 43.50 g/100 g of fat, respectively, while Aleppo pine and milk thistle seeds contain 29.8 and 19.3 g/100 g of fat. The high lipid content is consistent with studies highlighting the lipid-rich nature of seeds (63, 64), and pumpkin and sunflower seeds are recognized as excellent dietary fat sources.

The ash content results are higher than the 1.63% reported by Verma et al. (65) for pumpkin seed-based bars and the 1.32% reported by Umme et al. (66), indicating higher mineral content. Likely resulting from the inclusion of diverse seeds that are high in vital minerals including magnesium, phosphorus, zinc, potassium.

The variation in fiber content between the two cereal bars is likely due to ingredient differences that enhance dietary fiber, known for its numerous health benefits (67). Dietary fiber is acknowledged for its physiological and health promoting effects, including intestinal transit, glycemic control, and satiety, supporting its use in functional and health-oriented products (68). In the present study the high fiber level in both formulations can be explained by the use of diverse wild seeds notably milk thistle and Aleppo pin which naturally contribute insoluble fiber (69).

Furthermore, the addition of puffed rice may have influenced the overall fiber profile by enhacing the solid matrix and promoting the preservation of fibrous components in the product (70).

Similarly, the fiber content is above the 15.16 g/100 g reported by Verma et al. (65), which is attributed to the inclusion of a variety of seeds, such as milk thistle and Aleppo pine, along with puffed rice and other binders, enhancing the nutritional value of the formulation. While carbohydrates values are slightly lower than that reported by Sharma et al. (62) for a pumpkin seed-based bar, but are consistent with the values of Szydłowska et al. (71).

Finally, the calorific values obtained in the study are comparable to those found by Arruda et al. (30), who reported 422.61 kcal/100g for cereal bars made with almond and pineapple peels. According to Sharma et al. (72), cereal bar above 200 Kcal/100 g is classified as energy rich products, and the present formulation falls into this category. This high energy value is mainly explained by the incorporation of lipid rich ingredients such as sunflower butter. Overall, these findings underscore the distinct nutritional profiles of the two bars, offering a range of functional and dietary benefits.

Concerning the antioxidant properties, the results can be attributed to the ingredients used for the formulation, especially pomegranate molasses.

Pomegranate molasses is one of the main sources of phenolic compounds. According to Habib et al. (73), pomegranate is particularly rich in phenolic acids, hydrolyzable tannins, and flavonoids, which contribute significantly to the antioxidant potential of the products in which it is incorporated. Similarly, Kamal et al. (74) report that pomegranate molasses contains a significant amount of phenolic compounds and flavonoids, thereby enhancing its radical scavenging activity. In addition, the seeds included in the formulation also play an essential role. Several studies have shown that sunflower seeds are a beneficial source of phenolic compounds and have significant antioxidant activity (75–77). Pumpkin seeds are also known for their high content of flavonoids, phenolic compounds, and carotenoids, molecules widely documented for their ability to neutralize various reactive oxygen species (78, 79). Thus, the synergistic combination of these ingredients–pomegranate molasses, seeds rich in bioactive compounds, and puffed rice–explains the high content of phenolic compounds (TPC), flavonoids (TFC), and total antioxidant capacity measured in both bars.

The variation in sensory evaluation scores could be attributed to individual preferences attributes, which is common in in consumer based hedonic tests. Furthermore, the optimized balance of ingredients in both formulations may have improved the texture and overall acceptability (80).

In the present study, the higher acceptability observed for the first optimal bars could be explained by the combined sensory contribution of sunflower butter and pomegranate molasses. These ingredients provide natural sweetness, which is generally preferred by consumers. In addition, sunflower butter is rich in lipids, which may have improved mouthfeel and enhance flavor release, thereby positively influencing taste perception and overall acceptability (81).

### 4.3 Water activity and cold plasma treatment

The low water activity (a_w_) observed is consistent with typical low-moisture snack bars and supports microbiological stability during ambient storage. Water activity is a measure of the availability of unbound water for microbial growth and chemical reaction. An a_w_ value below 0.6 is generally considered effective for inhibiting the growth of most microorganisms (82). These results are similar to those of Babu and Mohan (83), who found a_w_ values ranging from 0.47 to 0.57 in cereal bars. In addition, Lu and Zhou (84) reported that protein-enriched cereal bars commonly show a_w_ values between 0.50 and 0.80. Similar observations were also described by Pallavi et al. (85). The measured a_w_ values of our gluten-free cereal bars (approximately 0.48) indicate limited water availability, suggesting that the products may be stored for extended periods with a reduced risk of microbial growth, mycotoxin production, and quality deterioration. These values fall within the typical range reported for dry cereal bars (0.10–0.60) (86, 87). By maintaining an a_w_ well below the critical threshold of 0.6, the developed bars are expected to effectively restrict the growth of common spoilage microorganisms and pathogens, including Listeria monocytogenes, while also limiting undesirable chemical and enzymatic reactions (58).

Furthermore, the current results are consistent with carob-enriched bars (a_w_ < 0.5), which have been reported to provide favorable stability condition for functional component such as probiotics. (87) and protein-based composite bars (~0.4) designed to counter lipid oxidation (88). Compared to date and cereal-based formulations with higher aw value (0.515–0.521) which may provide a larger risk of mold spoiling, our bars improved physicochemical stability (89). Ultimately, staying below the 0.6 is expected to enhance shelf life by inhibiting microbial growth and reducing undesirable chemical and enzymatic process that lead to quality degradation (58).

Immediately after treatment, cold plasma induced a reduction of approximately 0.6 log CFU/g in both formulations, corresponding to a decrease of more than 70% in viable cell counts. The slightly higher total aerobic count observed in optimal 1 compared to optimal 2 may be attributable to minor differences in ingredients or processing steps. Nevertheless, both values remained significantly below the levels reported in the literature as associated with microbial spoilage in dried fruit or vegetable matrices under various storage conditions (90, 91).

Yeast and mold counts were not detected in both treated and untreated optimal bars, indicating excellent fungal safety. The absence of yeasts and molds is particularly significant for low-moisture products, as these organisms represent the primary spoilage agents in such matrices due to their tolerance to reduced water activity. Similar studies on cereal-based snack products have reported that microbial counts remain stable or show only slight changes during the first weeks of storage when water activity is low and packaging conditions are adequate (92, 93).

Overall, these findings demonstrate that cold plasma treatment at 40 W for 20 min effectively reduced the aerobic mesophilic bacterial load in both cereal bar formulations. A slight increase in microbial counts was observed during storage; however, all values remained well below those reported in the literature as being associated with microbial spoilage in dried fruit or vegetable matrices under comparable storage conditions. The consistent maintenance of low microbial populations in plasma-treated samples throughout the 14-day storage period indicates sustained antimicrobial effectiveness of the cold plasma treatment.

## 5 Conclusions

This study demonstrates the feasibility of developing nutritious cereal bars with a desirable texture, extended shelf life, high antioxidants, higher microbial stability, and strong consumer acceptance by combining puffed rice, seeds, and binders. Optimization identified two optimal ingredient proportions, favoring crispness. The resulting bars are high in protein, fat, fiber, carbohydrates, phenolic content, and caloric value, making them nutritious, gluten-free, high-energy, and high-fiber snacks. The present study also showed, as a preliminary validation test, that the application of cold plasma treatment for 20 min during storage of the two optimized formulations contributed to a reduction in microbial load, confirming that treatment conditions previously reported in the literature may also be effective for our differently formulated cereal bar mixtures. These findings suggest that cold plasma treatment may represent a promising non-thermal technology for enhancing the microbiological safety of low-moisture cereal bars.

In addition, these findings underscore the potential of the optimized cereal bars as functional snacks that offer a convenient and delicious way to meet dietary needs and can be recommended as nutritious, gluten-free, high-energy, and high-fiber bars. Importantly, this study contributes to the field by highlighting the innovative use of diverse ingredients and optimization techniques in gluten-free cereal bar formulation. Based on these promising cold plasma preliminary results, future research will include a comprehensive shelf-life study of plasma-treated cereal bars to evaluate changes depend on packaging materials, storage conditions, physicochemical, nutritional, antioxidant, and sensory quality attributes, in order to identify the most suitable treatment conditions for industrial application.

## Data Availability

The original contributions presented in the study are included in the article/supplementary material, further inquiries can be directed to the corresponding author.
